# SEMA3G, downregulated by ncRNAs, correlates with favorable prognosis and tumor immune infiltration in kidney renal clear cell carcinoma

**DOI:** 10.18632/aging.205277

**Published:** 2023-12-08

**Authors:** Jian Yuan, Guorong Yuan

**Affiliations:** 1 Department of Medical Oncology, Longhua Hospital, Shanghai University of Traditional Chinese Medicine, Shanghai 200032, P.R. China; 2 Cancer Center, Department of Medical Oncology, Zhejiang Provincial People’s Hospital (Affiliated People’s Hospital), Hangzhou Medical College, Hangzhou, Zhejiang 310014, P.R. China

**Keywords:** SEMA3G, TBX2-AS1, miR-146a-5p, miR-146b-5p, kidney renal clear cell carcinoma (KIRC), prognosis, immune infiltration, noncoding RNA (ncRNA)

## Abstract

Kidney renal clear cell carcinoma (KIRC), relatively aggressive subtype of renal cell carcinoma, lacks of effective targets and promising biomarkers. Recently, although the function and immune correlation of semaphorin 3G (SEMA3G) in cancer draw more and more attention, its specific role and mechanism in KIRC are still not fully understood. In this work, we firstly conducted pan-cancer expression and survival bioinformatic analysis for SEMA3G and showed that SMEA3G might be a potential tumor suppressor and favorable prognostic biomarker in KIRC. Next, upstream noncoding RNA (ncRNA) regulatory mechanism of SEMA3G in KIRC was explored. By performing a series of *in silico* analyses, we identified that TBX2-AS1-miR-146a/b-5p axis was partially responsible for SEMA3G downregulation in KIRC. Furthermore, we also confirmed significant correlation of SEMA3G expression with tumor immune infiltration levels, expression of biomarkers of immune cells or immune checkpoints in KIRC. Taken together, the current data elucidated that ncRNA-caused downregulation of SEMA3G markedly linked to favorable prognosis and tumor immune infiltration in KIRC.

## INTRODUCTION

Renal cell carcinoma (RCC) is a cancer type occupying about 3% of all cancer cases, with an annual increase of 2% incidence, resulting in more than 400,000 new cases and 175,000 deaths in 2018 all over the world [1]. Kidney renal clear cell carcinoma (KIRC), a rather aggressive subtype, accounts for approximately 85% of metastatic RCC cases and 67% of all stage RCC [2]. Moreover, 25% to 30% of KIRC cases accompany metastases at diagnosis and 20% to 30% of these cases present relapse after undergoing surgery for local tumors [3]. Lacking of reliable therapeutic targets and stable prognostic markers bears responsibility for that. Therefore, it is extremely urgent need to seek and develop effective targets and promising biomarkers in KIRC.

Semaphores (SEMAs) was successively discovered to be as chemo-repulsive molecules for axonal growth cones and be involved in regulating cell motility in the context of vascular growth and tumor metastasis, which can be divided into two subgroups, consisting of transmembrane proteins (classes 1, 4, 6 and 7) and secretory proteins (classes 2 and 3) [4]. SEMA3G is a member of SEMAs. Only a few studies have been done in the field of SMEA3G’ function and mechanism in human cancer. Karayan et al. suggested that SEMA3G might be used as a prognostic marker in glial tumours [5]; Zhou et al. confirmed that SEMA3G possessed anti-migration and anti-invasion abilities in glioma cells [6]. In KIRC, two reports have together demonstrated that SEMA3G might act as a key component of immune-related prognostic signatures [7, 8]. However, a comprehensive investigation regarding the expression, role, mechanism and the association between SEMA3G and tumor immune infiltration is still absent.

In this study, we firstly performed a pan-cancer expression and survival analysis for SEMA3G in multiple human malignancies. Next, the upstream non-coding RNA (ncRNA) regulatory mechanism responsible for SEMA3G downregulation in KIRC was explored. Finally, we determined the correlation of SEMA3G expression with tumor immune infiltration level, biomarkers of immune cells or immune checkpoint levels in KIRC. Collectively, our current findings indicated that ncRNA-caused downregulation of SEMA3G linked to poor prognosis and tumor immune infiltration in KIRC.

## MATERIALS AND METHODS

### TCGA data download, process and analysis

The mRNA expression data of 18 types of human cancer, including BLCA, BRCA, CHOL, COAD, ESCA, GBM, HNSC, KICH, KIRC, KIRP, LIHC, LUAD, LUSC, PRAD, READ, STAD, THCA and UCEC, were downloaded from TCGA database (https://genome-cancer.ucsc.edu/). Next, these expression data were processed and normalized using R package limma [9], after which differential expression analysis was performed in these cancer types. *P*-value < 0.05 was considered as statistically significant.

### GEPIA database analysis

GEPIA (http://gepia.cancer-pku.cn/), a newly developed interactive web tool for analyzing the RNA sequencing expression data of 9,736 tumors and 8,587 normal samples from the TCGA and the GTEx projects [10], was employed to validate SEMA3G’s and lncRNAs’ expression in various types of human cancer. *P*-value < 0.05 was considered as statistically significant. GEPIA was also used to perform survival analysis for SEMA3G in human cancer. Logrank *P*-value < 0.05 was considered as statistically significant. Besides, expression relationship of SEMA3G-lncRNA or SEMA3G-immune checkpoint pairs in KIRC was also assessed by GEPIA database. |R| > 0.1 and *P*-value < 0.05 were set as selection criteria for identifying significant correlated pairs in KIRC.

### miRNA prediction

Seven online tools, consisting of PITA, RNA22, miRmap, microT, miRanda, PicTar and TargetScan, were introduced to predict the upstream possible miRNAs that could potentially target SEMA3G. For improving the analytic accuracy, only these miRNAs that commonly appeared in more than one prediction programs were included in this study. These predicted miRNAs were considered as candidate miRNAs of SEMA3G and a miRNA-SEMA3G network was established using Cytoscape software.

### starBase database analysis

starBase (http://starBase.sysu.edu.cn/) is a database for decoding miRNA-ceRNA, miRNA-ncRNA and protein-RNA interaction networks from CLIP-Seq data [11]. In this study, starBase database was employed to conduct miRNA-related correlation analysis and expression analysis in KIRC. Moreover, this database was also used to evaluate expression relationship of SEMA3G-lncRNA pairs and SEMA3G-immune checkpoint pairs in KIRC. *P*-value < 0.05 was considered as statistically significant.

### Kaplan-Meier plotter database analysis

Kaplan-Meier plotter (http://kmplot.com/analysis/) is an online database for assessing the effects of miRNAs or genes in more than 20 types of human cancer including KIRC. This tool was introduced to determine the prognostic values of miRNAs in KIRC. The survival plots were directly downloaded from the website. Logrank *P*-value < 0.05 was considered as statistically significant.

### lncRNA prediction and intersection analysis

The possible lncRNAs that could potentially bind to miR-146a-5p, miR-146b-5p or miR-589-5p were predicted by two online tools, consisting of starBase (http://starBase.sysu.edu.cn/) and miRNet (http://www.mirnet.ca/) database [11, 12]. Next, intersection analysis was performed to further obtain most potential binding lncRNAs that commonly appeared in the two prediction databases by VENNY2.1 (https://bioinfogp.cnb.csic.es/).

### TIMER database analysis

TIMER (http://cistrome.shinyapps.io/timer/), an online web server for integrated analysis of tumor infiltrating immune cells [13], was used to analyze calculate the immune cell infiltrating levels under various copy number of SEMA3G in KIRC. Furthermore, TIMER database was also to analyze the correlation of SEMA3G with various immune cells in KIRC. *P*-value < 0.05 was considered as statistically significant.

### Statistical analysis

The statistical analysis in this study was automatically calculated by these online databases or tools as mention above. *P*-value < 0.05 or logrank *P*-value < 0.05 was considered as statistically significant.

## RESULTS

### Pan-cancer analysis of SEMA3G’s expression landscape

**Figure 1:**
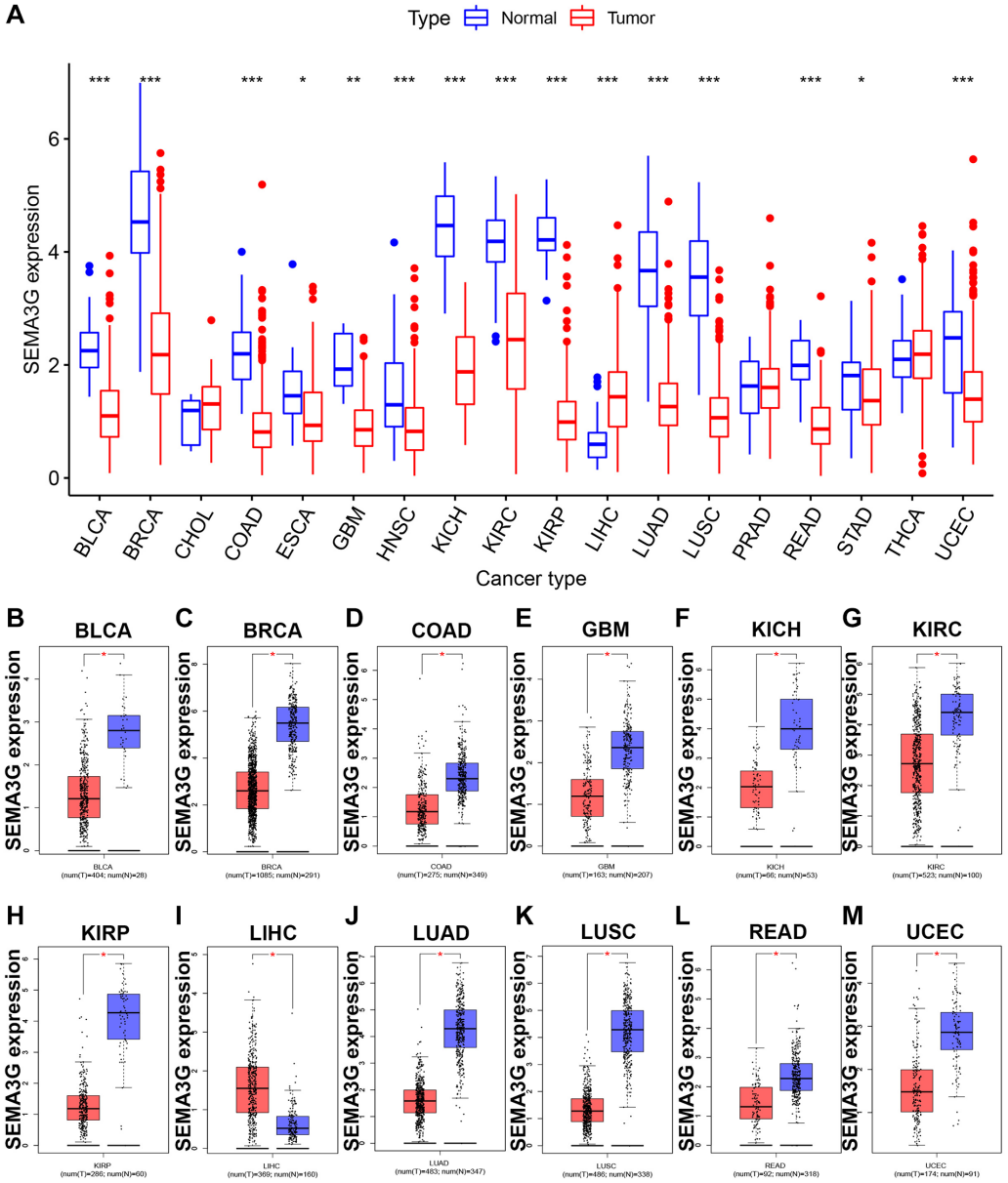
**The expression analysis for SEMA3G in multiple human malignancies.** (**A**) The expression of SEMA3G in 18 types of human cancer based on TCGA cancer and normal expression data. The expression levels of SEMA3G in TCGA BLCA (**B**), BRCA (**C**), COAD (**D**), GBM (**E**), KICH (**F**), KIRC (**G**), KIRP (**H**), LIHC (**I**), LUAD (**J**), LUSC (**K**), READ (**L**) and UCEC (**M**) tissues compared with corresponding TCGA and GTEx normal tissues. ^*^P < 0.05; ^**^P < 0.01; ^***^P < 0.001.

To explore the expression levels of SEMA3G in human cancer, a pan-cancer analysis was performed. By usage of TCGA cancer and normal data, we found that SEMA3G was significantly downregulated in most of cancer types, including BLCA, BRCA, COAD, ESCA, GBM, HNSC, KICH, KIRC, KIRP, LUAD, LUSC, READ, STAD and UCEC (Figure 1A). However, in LIHC, SEMA3G expression in cancer tissues was markedly increased when compared with normal tissues. In order to further validate the analytic result, GEPIA database was introduced, which contains TCGA and GTEx normal expression data. As shown in Figure 1B–1M, identical with the above result, SEMA3G was obviously decreased in BLCA, BRCA, COAD, ESCA, GBM, HNSC, KICH, KIRC, KIRP, LUAD, LUSC, READ, STAD and UCEC and upregulated in LIHC cancer tissues when compared with corresponding normal controls. These findings suggest that SEMA3G might act as a key regulator in carcinogenesis of these pointed human malignancies.

### Survival analysis of SEMA3G in human cancer

**Figure 2:**
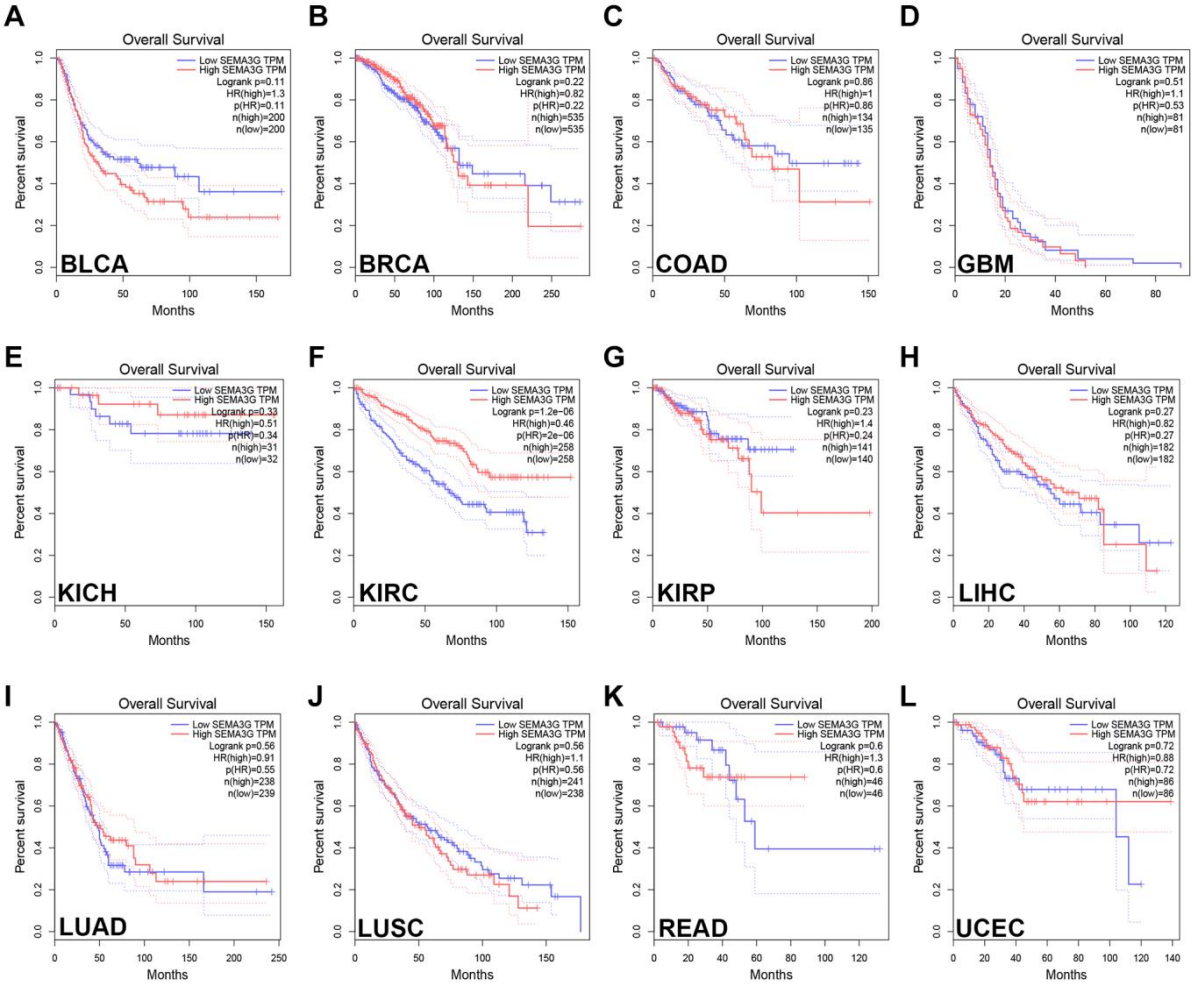
**The overall survival (OS) analysis for SEMA3G in different types of human cancer was determined by GEPIA database.** The OS plot of SEMA3G in BLCA (**A**), BRCA (**B**), COAD (**C**), GBM (**D**), KICH (**E**), KIRC (**F**), KIRP (**G**), LIHC (**H**), LUAD (**I**), LUSC (**J**), READ (**K**) and UCEC (**L**).

**Figure 3:**
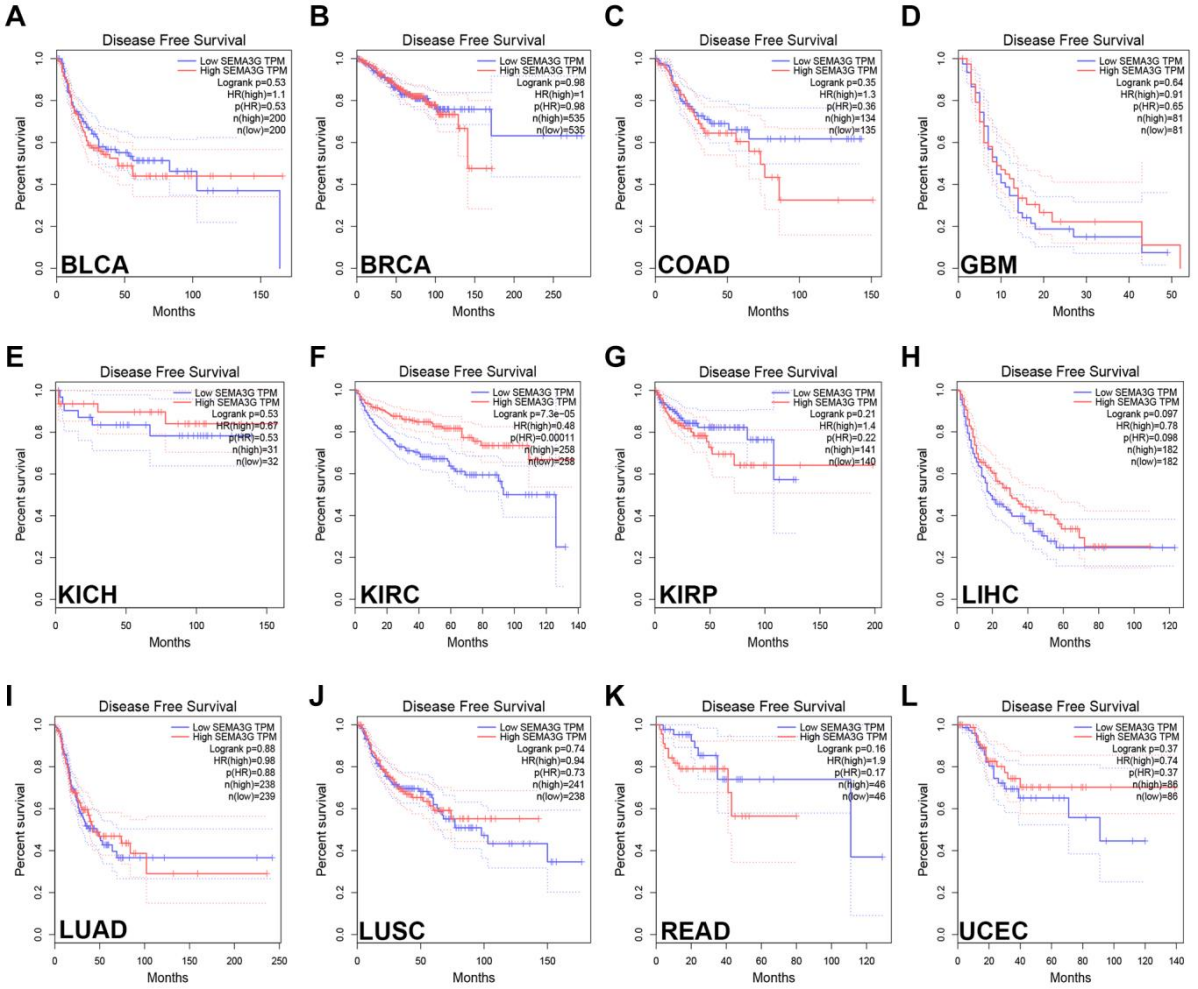
**The disease-free survival (RFS) analysis for SEMA3G in different types of human cancer was determined by GEPIA database.** The RFS plot of SEMA3G in BLCA (**A**), BRCA (**B**), COAD (**C**), GBM (**D**), KICH (**E**), KIRC (**F**), KIRP (**G**), LIHC (**H**), LUAD (**I**), LUSC (**J**), READ (**K**) and UCEC (**L**).

Next, the prognostic values of SEMA3G in BLCA, BRCA, COAD, ESCA, GBM, HNSC, KICH, KIRC, KIRP, LIHC, LUAD, LUSC, READ, STAD and UCEC was assessed. Two prognostic indices, consisting of overall survival (OS) and disease-free survival (RFS), was included. As presented in Figure 2, only KIRC patients with high expression of SEMA3G indicated favorable OS. Intriguingly, KIRC patients with higher expression of SEMA3G possessed better RFS as shown in Figure 3. No statistical significance of SEMA3G for predicting patients’ prognosis in other types of human cancer was observed. Taken together, SEMA3G might be a promising favorable prognostic biomarker in KIRC.

### Prediction and analysis of upstream miRNAs of SEMA3G in KIRC

**Figure 4:**
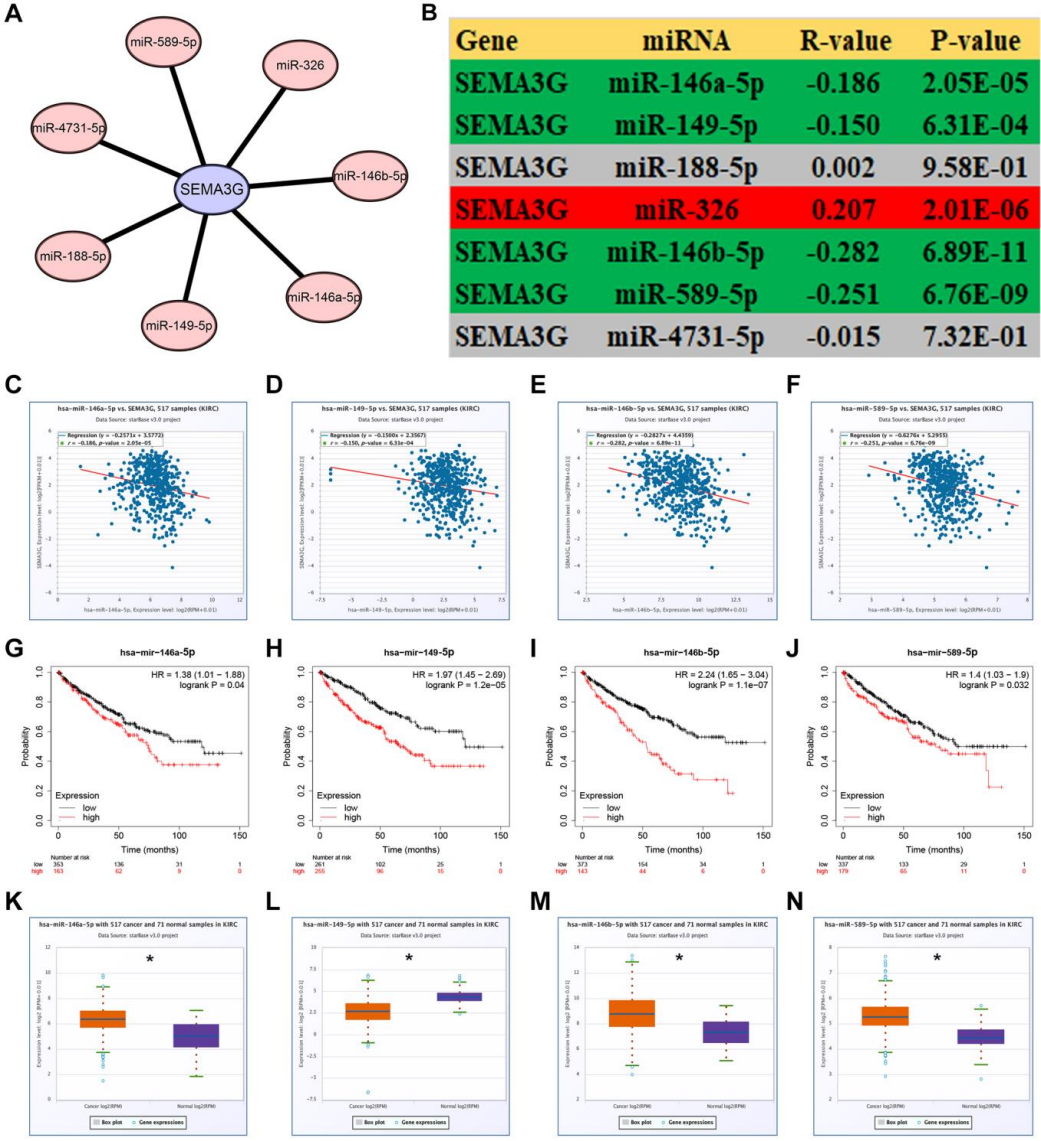
**Prediction and analysis of upstream miRNAs of SEMA3G in KIRC.** (**A**) The miRNA-SEMA3G regulatory network established by Cytoscape software. (**B**) The expression correlation between predicted miRNAs and SEMA3G in KIRC calculated by starBase database. The expression correlation of SEMA3G with miR-146a-5p (**C**), miR-149-5p (**D**), miR-146b-5p (**E**), or miR-589-5p (**F**) in KIRC. The prognostic values of miR-146a-5p (**G**), miR-149-5p (**H**), miR-146b-5p (**I**), or miR-589-5p (**J**) in KIRC. The expression levels of miR-146a-5p (**K**), miR-149-5p (**L**), miR-146b-5p (**M**), or miR-589-5p (**N**) in KIRC. ^*^P < 0.05.

It has been widely acknowledged that ncRNAs are involved in regulation of gene’s expression and functions. Thus, upstream miRNAs of SEMA3G were firstly predicted using a variety of online prediction tools, including PITA, RNA22, miRmap, microT, miRanda, PicTar and TargetScan (Table 1). At the end, 7 miRNAs were forecasted to potentially target SEMA3G. For better visualization, a miRNA-SEMA3G network was established through Cytoscape software (Figure 4A). Next, the expression relationship of SEMA3G with its predicted miRNAs in KIRC was evaluated using TCGA KIRC data (Figure 4B). As presented in Figure 4C–4F, 4 of 7 miRNAs, involving miR-146a-5p, miR-149-5p, miR-146b-5p and miR-589-5p, were significantly negatively correlated with SEMA3G expression in KIRC. Subsequently, survival analysis for the four miRNAs was conducted by Kaplan-Meier plotter. Intriguingly, high expression of all the four miRNAs indicated poor prognosis in KIRC (Figure 4G–4J). Finally, we also determined the expression levels of them in KIRC. The results showed that miR-146a-5p, miR-146b-5p and miR-589-5p were upregulated but miR-149-5p was downregulated in cancer tissues when compared with normal tissues (Figure 4K–4N). All these findings demonstrate that miR-146a-5p, miR-146b-5p and miR-589-5p might be the most potential upstream regulatory miRNAs of SEMA3G in KIRC.

**Table 1:** The potential miRNAs of SEMA3G predicted by online miRNA-target prediction tools.

**miRNA name**	**miR-146a-5p**	**miR-149-5p**	**miR-188-5p**	**miR-326**	**miR-146b-5p**	**miR-589-5p**	**miR-4731-5p**
PITA	1	1	1	1	1	1	
RNA22							
miRmap		1	1				1
microT	1		1	1	1	1	1
miRanda							
PicTar					1	1	
TargetScan	1				1		

### Prediction and analysis of upstream lncRNAs of miRNA in KIRC

**Figure 5:**
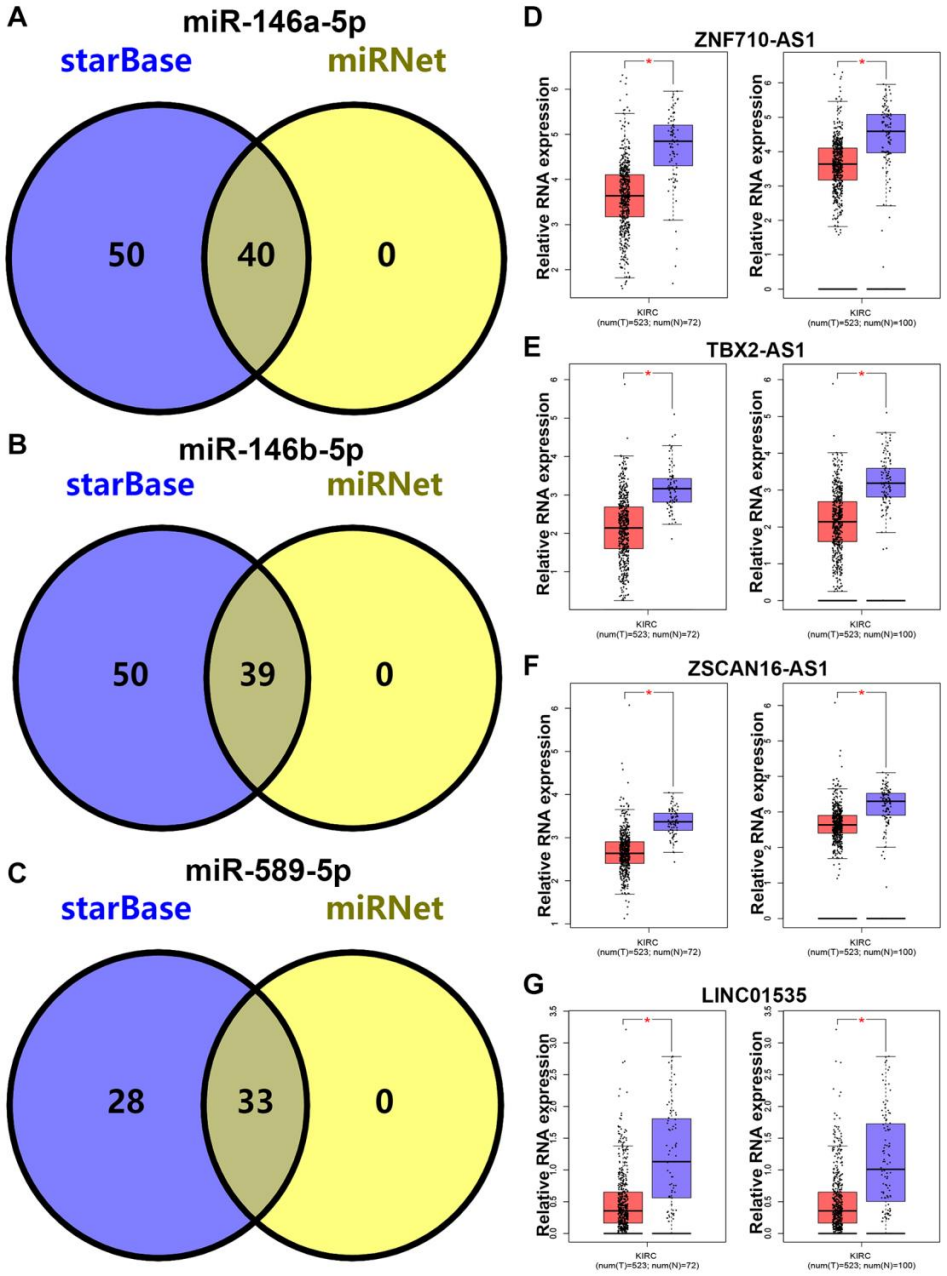
**Prediction and analysis for upstream lncRNAs of candidate miRNAs in KIRC.** The intersection analysis for lncRNAs of miR-146a-5p (**A**), miR-146b-5p (**B**) and miR-589-5p (**C**) predicted by starBase and miRNet databases. The expression levels of ZNF710-AS1 (**D**), TBX2-AS1 (**E**), ZSCAN16-AS1 (**F**) and LINC01535 (**G**) in KIRC compared with normal controls. ^*^P < 0.05.

**Figure 6:**
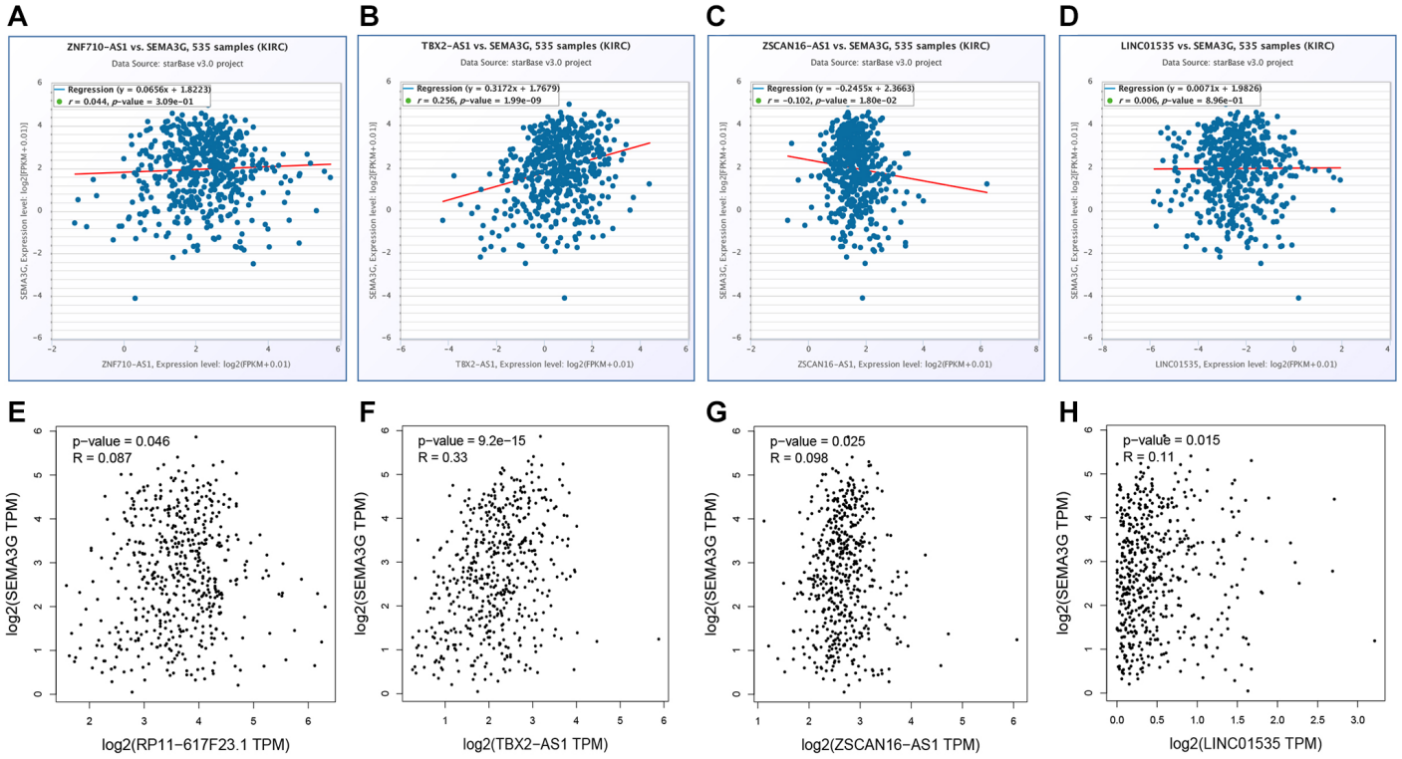
**The expression relationship of SEMA3G with candidate lncRNAs in KIRC.** The expression correlation of SEMA3G with ZNF710-AS1 (**A**), TBX2-AS1 (**B**), ZSCAN16-AS1 (**C**) or LINC01535 (**D**) in KIRC determined by starBase database. The expression correlation of SEMA3G with ZNF710-AS1 (**E**), TBX2-AS1 (**F**), ZSCAN16-AS1 (**G**) or LINC01535 (**H**) in KIRC determined by GEPIA database.

Subsequently, upstream potential lncRNAs of miR-146a-5p, miR-146b-5p and miR-589-5p were predicted. Two databases, consisting of starBase and miRNet, were employed. As shown in Figure 5A–5C, 40, 39 and 33 lncRNAs were respectively found to potentially bind to miR-146a-5p, miR-146b-5p and miR-589-5p. Next, the expression correlation of the three miRNAs with their corresponding lncRNAs was assessed in KIRC. As listed in Supplementary Table 1, six lncRNAs were negatively correlated with miR-146a-5p, including EPB41L4A-AS1, SNHG7, SLC25A21-AS1, TBX2-AS1 and LINC00665. For miR-146a-5p, there were also several negatively correlated lncRNAs in KIRC, consisting of MIR4453HG, EPB41L4A-AS1, ZSCAN16-AS1, HCG18, LINC02538, EBLN3P, LINC00963, SNHG7, CCDC183-AS1, NEAT1, SLC25A21-AS1, LINC02288, ZNF710-AS1, TBX2-AS1, LINC00665, LINC01535 and XIST (Supplementary Table 2). As presented in Supplementary Table 3, miR-589-5p was significantly inversely linked to LINC01128 and ZFAS1 in KIRC. Followingly, expression levels of the 19 lncRNAs in KIRC were detected by using TCGA cancer data, TCGA normal data and GTEx normal data. The results demonstrated that 4 of 19 lncRNAs, including ZNF710-AS1, TBX2-AS1, ZSCAN16-AS1 and LINC01535, were markedly downregulated in KIRC cancer tissues when compared with normal tissues (Figure 5D–5G). Finally, we assessed the expression correlation of the four lncRNAs with SEMA3G in KIRC. As presented in Figure 6A–6H, SEMA3G was significantly positively correlated with TBX2-AS1 in KIRC, which was in accordance with the ceRNA hypothesis. Collectively, TBX2-AS1 was the potential upstream regulatory lncRNA of miR-146a-5p/miR-146b-5p-SEMA3G axis in KIRC.

### SEMA3G positively correlates with immune cell infiltration in KIRC

**Figure 7:**
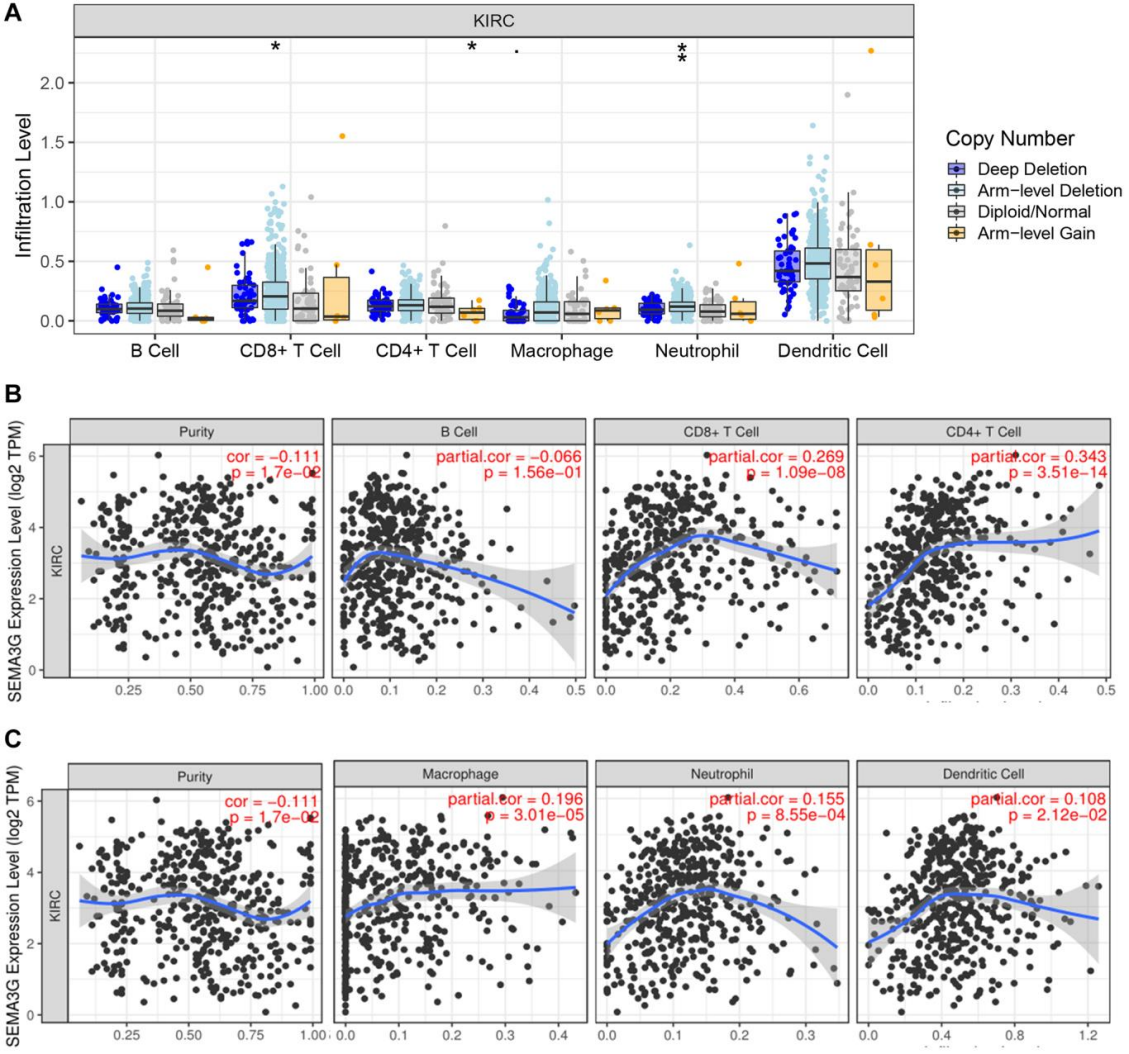
**The relationship of immune cell infiltration with SEMA3G level in KIRC.** (**A**) The infiltration level of different immune cells under various copy numbers of SEMA3G in KIRC. (**B**) The correlation of SEMA3G expression level with B cell, CD8^+^ T cell or CD4^+^ T cell in KIRC. (**C**) The correlation of SEMA3G expression level with macrophage, neutrophil or dendritic cell in KIRC. ^*^P < 0.05; ^**^P < 0.01.

Class 3 semaphorins is closely linked to immune system and correlates with tumor immune infiltration. To ascertain if SEMA3G is associated with immune cell infiltration in KIRC, TIMER database was employed. As presented in Figure 7A, CD8^+^ T cell and neutrophil infiltration levels were obviously increased under arm-level deletion of SEMA3G’s copy number in KIRC and CD4^+^ T cell infiltration level was markedly decreased under arm-level gain of SEMA3G’s copy number in KIRC. SEMA3G expression level was significantly positively correlated with CD8^+^ T cell or CD4^+^ T cell infiltration level in KIRC (Figure 7B). No statistical correlation of SEMA3G with B cell infiltration level in KIRC was observed. Moreover, as shown in Figure 7C, SEMA3G expression was markedly positively associated with macrophage, neutrophil or dendritic cell infiltration level in KIRC.

### Expression correlation of SEMA3G with biomarkers of immune cells in KIRC

In order to further probe the role of SEMA3G in tumor immune infiltration, we analyzed the expression correlation of SEMA3G with biomarkers of immune cells in KIRC determined by GEPIA database. As listed in Table 2, SEMA3G’s expression level was statistically positively correlated with CD4^+^ T cell’s biomarker (CD4), M1 macrophage’s biomarker (NOS2 and PTGS2), M2 macrophage’s biomarker (CD163, VSIG4 and MS4A4A), neutrophil’s biomarker (ITGAM and CCR7) and dendritic cell’s biomarker (HLA-DPB1, HLA-DRA, HLA-DPA1, CD1C and NRP1) in KIRC. However, no significant expression correlation of SEMA3G’s expression with biomarkers of B cell in KIRC was observed. These findings were in accordance with the analytic result from correlation analysis between SEMA3G and tumor immune infiltration. Taken together, SEMA3G might be positively correlated with immune cell infiltration in KIRC, especially CD4^+^ T cell, macrophage, neutrophil and dendritic cell.

**Table 2:** Correlation analysis between SEMA3G and biomarkers of immune cells in KIRC determined by GEPIA database.

**Immune cell**	**Biomarker**	***R*-value**	***P*-value**
B cell	CD19	−0.05	0.26
	CD79A	−0.02	0.68
CD8+ T cell	CD8A	−0.00	0.96
	CD8B	0.02	0.73
CD4+ T cell	CD4	**0.20**	0.00^**^
M1 Macrophage	NOS2	**0.67**	0.00^**^
	IRF5	−0.07	0.10
	PTGS2	**0.16**	0.00^**^
M2 Macrophage	CD163	**0.16**	0.00^**^
	VSIG4	**0.11**	0.01^*^
	MS4A4A	**0.18**	0.00^**^
Neutrophil	CEACAM8	0.08	0.09
	ITGAM	**0.16**	0.00^**^
	CCR7	**0.18**	0.00^**^
Dendritic cell	HLA-DPB1	**0.17**	0.00^**^
	HLA-DQB1	0.04	0.33
	HLA-DRA	**0.12**	0.01^*^
	HLA-DPA1	**0.16**	0.00^**^
	CD1C	**0.29**	0.00^**^
	NRP1	**0.62**	0.00^**^
	ITGAX	−0.06	0.19

^*^P-value < 0.05; ^**^P-value < 0.01. The bold values indicate that these results are statistically significant.

### Relationship between SEMA3G and immune checkpoints in KIRC

**Figure 8:**
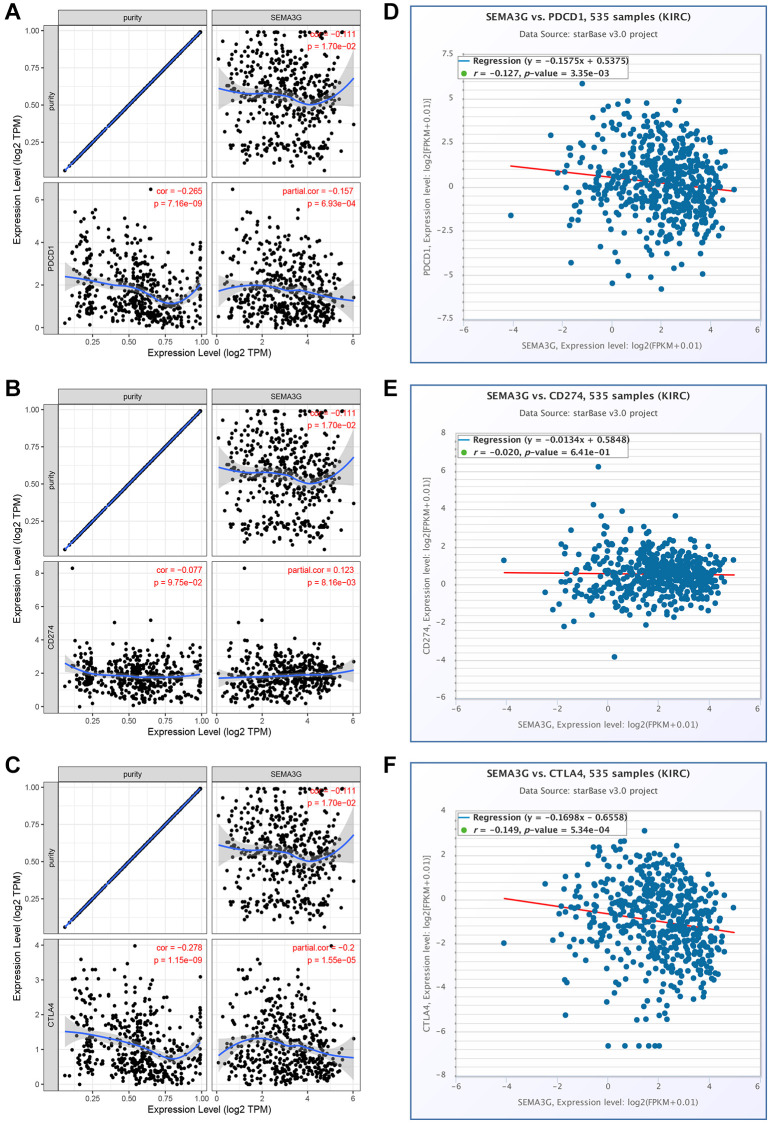
**Correlation of SEMA3G expression with PD-1, PD-L1 or CTLA-4 expression in KIRC.** Spearman correlation of SEMA3G with expression of PD-1 (**A**), PD-L1 (**B**) or CTLA-4 (**C**) in KIRC adjusted by purity using TIMER database. The expression correlation of SEMA3G with PD-1 (**D**), PD-L1 (**E**) or CTLA-4 (**F**) in KIRC validated by starBase database.

Considering the close relationship of SEMA3G with tumor immune infiltration in KIRC, we further assessed the correlation of SEMA3G expression with the levels of immune checkpoints (PD-1, PD-L1 and CTLA4). As shown in Figure 8A–8C, by analyzing TIMER database, SEMA3G expression was significantly negatively correlated with PD-1 or CTLA-4 level but was statistically positively associated with PD-L1 level in KIRC when was adjusted by purity. For further improving analytic accuracy, starBase database was also employed to evaluate expression correlation of SEMA3G with immune checkpoints in KIRC. As presented in Figure 8D–8F, SEMA3G expression was also markedly negatively linked to PD-1 or CTLA-4 level in KIRC. However, no significant expression correlation of SEMA3G with PD-L1 in KIRC was observed. It has been widely acknowledged that immune checkpoints are responsible for tumor immune escape. Thus, our present findings suggest that SEMA3G might be involved in the immune scape of KIRC.

## DISCUSSION

KIRC is a relatively aggressive subtype of RCC, which partially attributes to the lack of effective therapeutic choices and indicative prognostic biomarkers. Thus, it makes sense to study the molecular mechanism of KIRC pathogenesis and progression.

In this study, we firstly determined the expression levels and prognostic values of SEMA3G in multiple human malignancies by integration of TCGA and GTEx cancer and normal expression and survival data. The result suggested that SEMA3G was significantly downregulated in KIRC compared with normal controls and its high expression indicated favorable overall survival and disease-free survival of KIRC. To date, the role of SEMA3G in KIRC has not been detected and needs to be further explored. However, SEMA3G was reported to exert anti-tumor effects in migration and invasion of glioma [6]. Therefore, SEMA3G might be a potential tumor suppressor and favorable prognostic biomarker in KIRC.

NcRNAs, including miRNAs and lncRNAs, are involved in regulation of gene expression and functions in cancer [14, 15]. To ascertain if ncRNAs are responsible for SEMA3G downregulation in KIRC, upstream miRNAs were firstly predicted. According to the action mechanism of miRNA in modulating gene expression [16], there should be negative expression correlation of SEMA3G with predicted miRNAs in KIRC. Among all the miRNA-SEMA3G pairs, 4 miRNAs, consisting of miR-146a-5p, miR-149-5p, miR-146b-5p and miR-589-5p, were statistically inversely correlated with SEMA3G in KIRC. Subsequent analyses regarding survival and expression revealed that miR-146a-5p, miR-146b-5p and miR-589-5p were significantly upregulated and indicated poor prognosis in KIRC. Thus, the three miRNAs might be the most potential regulatory upstream miRNAs of SEMA3G in KIRC.

Next, the potential binding lncRNAs of the three miRNAs were predicted by starBase and miRNet databases [11, 12]. By combination of a series of bioinformatic analyses, TBX2-AS1 was identified as the most potential upstream binding lncRNA of miR-146a/b-5p-SEMA3G axis in KIRC. Rothzerg et al. showed that TBX2-AS1 was obviously upregulated in osteosarcoma [17]. TBX2-AS1 was also found to be an important component of prognostic signature for renal cell carcinoma patients with stage IV and histological grade G4 [18]. In this study, TBX2-AS1 was significantly downregulated and was markedly positively correlated with SEMA3G in KIRC. Taken together, TBX2-AS1-miR-146a/b-5p axis was a key upstream regulatory pathway of SEMA3G in KIRC.

Several studies have demonstrated that there is a close relationship between SEMA3G and inflammation or immunity. Ishibashi et al. suggested that SEMA3G could protect glomerular podocyte from lipopolysaccharide-caused inflammation [19]. Ji et al. showed that SEMA3G was an immune-related gene which played an important role in predicting prognosis of testicular germ cell tumor [20]. Moreover, Wan et al. confirmed that SEMA3G was also an immune-related gene in KIRC and possessed the prognostic value in KIRC [7]. Besides, by immunogenomic landscape analysis, the team of Gao Xin revealed that SEMA3G was a key component of a four immune-related genes signature in KIRC [8].

However, the correlation of SEMA3G with tumor immune infiltration in KIRC has not been determined in KIRC. Tumor immune cell infiltration plays important roles in influencing therapeutic efficacies of chemotherapy, radiotherapy or immunotherapy [4, 21]. Our current analytic result indicated that SEMA3G was positively correlated with tumor immune cell infiltration in KIRC, especially CD4^+^ T cell, macrophage, neutrophil and dendritic cell. Additionally, the expression levels of immune checkpoints also change the effects of immunotherapy [22]. Correlation analysis revealed a significant negative correlation of SEMA3G expression with PD-1 or CTLA-4 level in KIRC. All these data suggested that SEMA3G was statistically linked to tumor immune infiltration or immune checkpoints in KIRC, which might provide key clues for improving the efficacy of immunotherapy in KIRC in the future.

**Figure 9:**
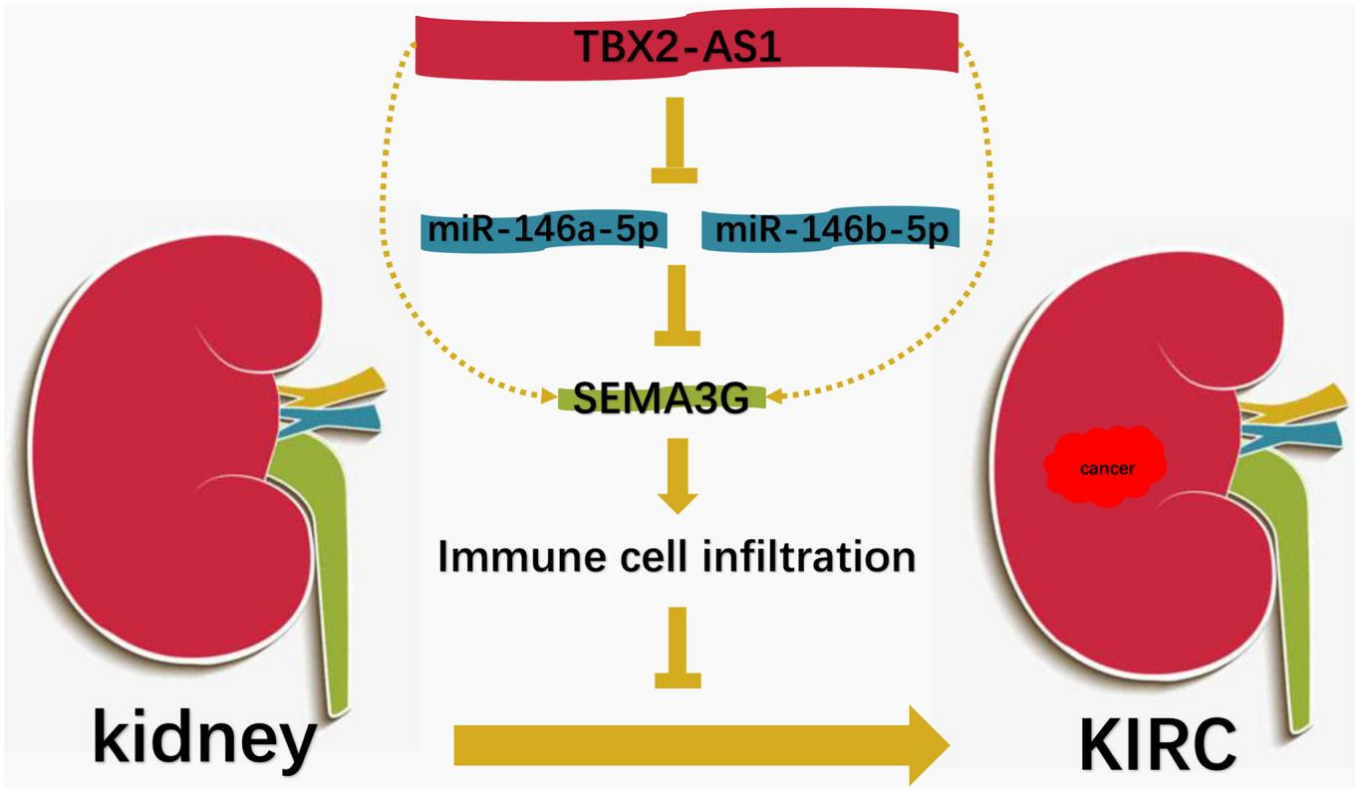
The model of TBX2-AS1-miR-146a/146b-5p-SEMA3G axis in KIRC.

Taken together, by performing a series of *in silico* analyses, we showed that SEMA3G was significantly downregulated in KIRC and possessed favorable prognosis of patients with KIRC, and identified that TBX2-AS1-miR-146a/b-5p pathway, an upstream axis of SEMA3G, was partially responsible for SEMA3G downregulation in KIRC (Figure 9). Furthermore, SEMA3G was markedly linked to tumor immune infiltration and expression of immune checkpoints in KIRC. However, there are some limitations in this work. For example, the results from this work were based on bioinformatic analysis; lack of the downstream action mechanism exploration of SEMA3G in KIRC. Thus, the current findings need to be further validated by much more basic experiments and clinical trials in the future.

## Supplementary Materials

Supplementary Tables
