# Supplementary material for: SEMA3G, downregulated by ncRNAs, correlates with favorable prognosis and tumor immune infiltration in kidney renal clear cell carcinoma
Source: Aging (Albany NY). 2023 Dec 8;15(23):13944–60. doi: 10.18632/aging.205277 (PMC10756116; doi:10.18632/aging.205277)
Supplement: Supplementary Tables [file aging-15-205277-s001.pdf]

SUPPLEMENTARY TABLES

Supplementary Table 1. The expression correlation of miR-146a-5p with predicted lncRNAs in KIRC determined by starBase.

miRNA	lncRNA	*R*-value	*P*-value
miR-146a-5p	LINC00115	0.101	2.19E-02
miR-146a-5p	MIR137HG	0.071	1.09E-01
miR-146a-5p	MIR181A1HG	0.195	7.74E-06
miR-146a-5p	MIR29B2CHG	0.046	3.00E-01
miR-146a-5p	ITPKB-IT1	0.250	8.63E-09
miR-146a-5p	MIR4453HG	0.031	4.87E-01
miR-146a-5p	TMEM161B-AS1	−0.028	5.19E-01
miR-146a-5p	**EPB41L4A-AS1**	**−0.161**	**2.43E-04**
miR-146a-5p	LINC00847	0.086	5.04E-01
miR-146a-5p	ZSCAN16-AS1	−0.077	8.21E-02
miR-146a-5p	HCG18	0.121	6.03E-03
miR-146a-5p	LINC02538	0.056	2.03E-01
miR-146a-5p	LINC01176	0.167	1.30E-04
miR-146a-5p	STAG3L5P-PVRIG2P-PILRB	0.075	8.92E-02
miR-146a-5p	CASC9	0.019	6.62E-01
miR-146a-5p	AZIN1-AS1	−0.049	2.67E-01
miR-146a-5p	MINCR	0.001	9.89E-01
miR-146a-5p	EBLN3P	0.024	5.85E-01
miR-146a-5p	FAM201A	−0.022	6.12E-01
miR-146a-5p	LINC00963	−0.003	9.40E-01
miR-146a-5p	**SNHG7**	**−0.214**	**9.46E-07**
miR-146a-5p	CCDC183-AS1	0.092	3.75E-02
miR-146a-5p	OLMALINC	−0.051	2.46E-01
miR-146a-5p	KCNQ1OT1	0.110	1.20E-02
miR-146a-5p	NEAT1	0.004	9.26E-01
miR-146a-5p	MALAT1	0.123	5.27E-03
miR-146a-5p	MIR100HG	0.105	1.70E-02
miR-146a-5p	LINC00567	0.036	4.08E-01
miR-146a-5p	**SLC25A21-AS1**	**−0.137**	**1.79E-03**
miR-146a-5p	LINC02288	0.063	1.53E-01
miR-146a-5p	**ZNF710-AS1**	**−0.170**	**1.03E-04**
miR-146a-5p	ZNF213-AS1	0.095	3.08E-02
miR-146a-5p	CRNDE	0.117	7.77E-03
miR-146a-5p	**TBX2-AS1**	**−0.101**	**2.18E-02**
miR-146a-5p	SNHG16	0.114	9.54E-03
miR-146a-5p	**LINC00665**	**−0.163**	**1.99E-04**
miR-146a-5p	LINC01535	−0.082	6.38E-02
miR-146a-5p	ZNF337-AS1	0.093	3.48E-02
miR-146a-5p	LINC01311	0.006	8.92E-01
miR-146a-5p	XIST	0.077	8.01E-02

The bold values indicate that these results are statistically significant.

Supplementary Table 2. The expression correlation of miR-146b-5p with predicted lncRNAs in KIRC determined by starBase.

miRNA	lncRNA	*R*-value	*P*-value
miR-146b-5p	LINC00115	−0.021	6.36E-01
miR-146b-5p	MIR137HG	0.121	5.83E-03
miR-146b-5p	MIR181A1HG	0.026	5.58E-01
miR-146b-5p	MIR29B2CHG	−0.070	1.12E-01
miR-146b-5p	ITPKB-IT1	0.002	9.59E-01
miR-146b-5p	**MIR4453HG**	**−0.194**	**9.06E-06**
miR-146b-5p	TMEM161B-AS1	−0.012	7.93E-01
miR-146b-5p	**EPB41L4A-AS1**	**−0.193**	**9.61E-06**
miR-146b-5p	LINC00847	−0.026	5.49E-01
miR-146b-5p	**ZSCAN16-AS1**	**−0.143**	**1.08E-03**
miR-146b-5p	**HCG18**	**−0.117**	**7.80E-03**
miR-146b-5p	**LINC02538**	**−0.226**	**2.05E-07**
miR-146b-5p	LINC01176	−0.016	7.13E-01
miR-146b-5p	CASC9	0.031	4.85E-01
miR-146b-5p	AZIN1-AS1	−0.051	2.46E-01
miR-146b-5p	MINCR	−0.039	3.74E-01
miR-146b-5p	**EBLN3P**	**−0.243**	**2.29E-08**
miR-146b-5p	FAM201A	0.010	8.23E-01
miR-146b-5p	**LINC00963**	**−0.303**	**2.04E-12**
miR-146b-5p	**SNHG7**	**−0.322**	**5.73E-14**
miR-146b-5p	**CCDC183-AS1**	**−0.150**	**6.43E-04**
miR-146b-5p	OLMALINC	−0.091	3.77E-02
miR-146b-5p	KCNQ1OT1	−0.046	2.96E-01
miR-146b-5p	**NEAT1**	**−0.154**	**4.23E-04**
miR-146b-5p	MALAT1	−0.037	3.98E-01
miR-146b-5p	MIR100HG	0.100	2.32E-02
miR-146b-5p	LINC00567	0.053	2.29E-01
miR-146b-5p	**SLC25A21-AS1**	**−0.270**	**4.19E-10**
miR-146b-5p	**LINC02288**	**−0.189**	**1.58E-05**
miR-146b-5p	**ZNF710-AS1**	**−0.269**	**5.33E-10**
miR-146b-5p	ZNF213-AS1	−0.090	4.07E-02
miR-146b-5p	CRNDE	0.198	5.88E-06
miR-146b-5p	**TBX2-AS1**	**−0.308**	**7.74E-13**
miR-146b-5p	SNHG16	0.127	3.74E-03
miR-146b-5p	**LINC00665**	**−0.136**	**1.90E-03**
miR-146b-5p	**LINC01535**	**−0.237**	**5.03E-08**
miR-146b-5p	ZNF337-AS1	0.080	7.01E-02
miR-146b-5p	LINC01311	−0.056	2.02E-01
miR-146b-5p	**XIST**	**−0.114**	**9.69E-03**

The bold values indicate that these results are statistically significant.

Supplementary Table 3. The expression correlation of miR-589-5p with predicted lncRNAs in KIRC determined by starBase.

miRNA	lncRNA	*R*-value	*P*-value
miR-589-5p	**LINC01128**	**−0.152**	**5.11E-04**
miR-589-5p	LINC01134	0.240	3.35E-08
miR-589-5p	RNF144A-AS1	−0.083	5.98E-02
miR-589-5p	TTN-AS1	0.138	1.72E-03
miR-589-5p	SH3BP5-AS1	0.096	2.87E-02
miR-589-5p	ZBTB11-AS1	0.093	3.36E-02
miR-589-5p	LINC00886	0.101	2.11E-02
miR-589-5p	LINC00887	−0.033	4.55E-01
miR-589-5p	HCG18	0.027	5.33E-01
miR-589-5p	SNHG15	0.042	3.42E-01
miR-589-5p	OR2A1-AS1	−0.032	4.63E-01
miR-589-5p	EBLN3P	−0.032	4.65E-01
miR-589-5p	LINC00963	0.139	1.58E-03
miR-589-5p	CCDC183-AS1	0.175	6.14E-05
miR-589-5p	ENTPD1-AS1	0.076	8.47E-02
miR-589-5p	KCNQ1OT1	0.115	8.67E-03
miR-589-5p	MALAT1	0.203	3.19E-06
miR-589-5p	HELLPAR	0.170	1.03E-04
miR-589-5p	LINC00943	0.108	1.42E-02
miR-589-5p	TPT1-AS1	−0.046	2.96E-01
miR-589-5p	ZFHX2-AS1	0.103	1.88E-02
miR-589-5p	ACTN1-AS1	0.068	1.24E-01
miR-589-5p	OIP5-AS1	−0.008	8.59E-01
miR-589-5p	LOXL1-AS1	0.029	5.13E-01
miR-589-5p	MAP3K14-AS1	0.100	2.32E-02
miR-589-5p	LINC00482	0.053	2.27E-01
miR-589-5p	LINC00665	−0.085	5.35E-02
miR-589-5p	LIPE-AS1	0.003	9.51E-01
miR-589-5p	A1BG-AS1	−0.077	8.11E-02
miR-589-5p	**ZFAS1**	**−0.171**	**8.89E-05**
miR-589-5p	LINC01547	−0.003	9.42E-01
miR-589-5p	MCM3AP-AS1	0.113	1.02E-02
miR-589-5p	MORC2-AS1	0.133	2.36E-03

The bold values indicate that these results are statistically significant.
